# Deep learning optimisation for cardiology: Neural Architecture Search-driven arrhythmia classification with electrocardiograms

**DOI:** 10.1371/journal.pone.0362129

**Published:** 2026-11-06

**Authors:** Erik Vanegas Müller, Arese Joe-Oshodi, Abhirup Banerjee, Mauricio Villarroel

**Affiliations:** 1 Department of Engineering Science, The Podium Institute for Sports Medicine and Technology, University of Oxford, Oxford, United Kingdom; 2 Department of Engineering Science, Institute of Biomedical Engineering, University of Oxford, Oxford, United Kingdom; Baruch Padeh Medical Center Poriya, ISRAEL

## Abstract

Cardiovascular disease is the leading cause of death worldwide. Sudden cardiac death (SCD) accounts for roughly 50% of all cardiac deaths. The electrocardiogram (ECG) is widely used for diagnosing cardiac disease. However, the complexity of interpretation limits the ECG’s efficacy. Modern deep learning methods can assist clinicians in diagnosis. We applied Neural Architecture Search (NAS) to identify optimal deep learning architectures for classifying cardiac arrhythmias with ECGs. We applied the Differentiable Architecture Search strategy to an AutoFormer search and trained, validated, and tested the resulting model on the PhysioNet Challenge 2021 (PC21) dataset (n = 88,253 from three continents). We optimised hyperparameters on the NAS output, exploring input patch size, class weighting, and loss function. The PC21 metric and the area under the receiver operating characteristic curve (AUROC) are the performance metrics. The NAS converged towards minimal architectural configurations (embedding dimension: 384, depth: 4, self-attention heads: 4, MLP ratio: 1) with a validation and test PC21 metric of 0.66 (PC21 winner: 0.63). The NAS-created network achieved an AUROC of 0.97 and a PC21 metric of 0.72 during testing (PC21 winner: 0.69). We tested the PC21 winner architecture (1D-convolutional neural network with attention mechanism) without the attention mechanism and with the NAS-suggested architecture of 4 self-attention heads (instead of 8). The architecture without the attention mechanism performed similarly to the one with 8 self-attention heads (AUROC: 0.90, PC21 metric: 0.69). The architecture with 4 self-attention heads scored an AUROC of 0.91 and improved the PC21 metric to 0.71. The attention mechanism visualisation for two SCD-related arrhythmias (premature ventricular contractions and prolonged QT-interval) showed more clinically relevant attention for the 4-self-attention-head architectures. We interpret architectural simplicity as driving performance in arrhythmia classification. The self-attention mechanism can autonomously abstract clinical representations, simplifying clinical deployment by eliminating the need for an explicit feature-extraction pipeline.

## Introduction

Cardiovascular disease is the leading cause of death worldwide, annually accounting for approximately 20.5 million deaths [1]. Cardiovascular deaths are expected to rise to 35.6 million in 2050 [1]. Sudden cardiac death (SCD) is defined as a death within one hour of the onset of symptoms. Presumably of cardiac origin, SCD accounts for roughly 50% of all cardiac deaths worldwide [2]. The largest absolute number of SCD cases occurs in the general population, which is unaware of its cardiac risk [2].

An electrocardiogram measures the heart’s electrical activity, allowing the identification of electrical abnormalities that can lead to life-threatening arrhythmias. These abnormalities predict early and late SCD events in middle-aged subjects [3]. Examples of severe electrical abnormalities are premature ventricular contractions (PVCs) and prolonged QT intervals (LQT). A PVC is a premature occurrence of an abnormal QRS complex without preceding P-wave [4]. PVCs can transition a ventricular tachycardia in sinus rhythm into a life-threatening rhythm called ventricular fibrillation [5]. A QT interval is prolonged if it is longer than 460 ms in women or 450 ms in men [6]. LQTs can develop into a dangerous arrhythmia called Torsades de Pointes [7]. In a study of 12,241 participants (54 ± 5.7 years; 26% black; 55% women), QT prolongation was associated with a 49% increased risk of SCD [8].

The clinical interpretation and diagnosis of cardiac abnormalities on the ECG are both time-consuming and require highly trained clinicians [9]. Over 100 different types of cardiac abnormalities (such as PVCs and LQTs) exist, with varying degrees of difficulty in identification [10]. Diagnostic algorithms aid clinicians in interpreting ECGs. These algorithms can be inaccurate in terms of sensitivity and specificity, lack diagnostic robustness, and vary in their evaluation of ECG recordings [11]. Therefore, diagnostic algorithms can introduce diagnostic uncertainty, delay treatment, and hamper clinicians’ assessment [9,11].

Compared to traditional algorithms, ECG-based deep learning models have shown significant improvements in interpretative accuracy and diagnostic robustness [12]. The European Heart Rhythm Association of the European Society of Cardiology (ESC), the Heart Rhythm Society, and the ESC Working Group on E-Cardiology identified AI research in SCD as a key research area in AI applied to Clinical Electrophysiology (the other being AI in atrial fibrillation management and AI in the electrophysiology lab) [13].

Transformers are a popular choice of deep learning models, which introduced the “self-attention” concept [14]. Originally proposed for a translation task, self-attention assigns each element of a sequence a query, key, and value. Multi-head self-attention mechanisms therefore allow for simultaneous focus on different relationships within a sequence. The number of attention heads is a central architectural parameter for transformer performance [15]. The architecture’s design is problem-dependent; it depends on the researchers’ prior knowledge and experience [16,17]. Other important transformer parameters include the embedding dimension, the number of encoders, and the multi-layer perceptron (MLP) ratio [18].

The design choices for deep learning architectures are not necessarily interchangeable across different applications. The field of neural architecture search (NAS) aims to automate the construction of deep learning architectures, thereby reducing potential biases introduced by traditional manual architecture design and hyperparameter fine-tuning [17,19]. So et al. [20] used an evolutionary NAS to discover a Transformer variant that outperformed the original model on a machine translation task. Chen et al. [18] applied a one-shot NAS to Vision Transformers, finding architectures that outperformed original model designs while using fewer parameters. Wu et al. [21] applied a differentiable NAS to Vision Transformers, thereby reducing parameter count while outperforming the original deep learning model. Azarmehr et al. [22] designed a NAS to classify anatomical echocardiographic views, reducing trainable parameters by up to 99.9% while maintaining comparable classification performance.

Most NAS approaches have been developed and validated on image classification tasks, with limited exploration of physiological time-series data such as ECGs. This is especially true for the search space, which contains the set of parameters the search strategy can choose from. Constructing a dedicated search space tailored to ECG signals is costly, time-consuming, and requires substantial domain expertise. We apply NAS to an existing image search space as a proof of concept to identify an optimal deep learning architecture for arrhythmia classification. We hypothesise that automated architecture search identifies architectures with superior or comparable classification performance, replacing costly manual trial-and-error with a single optimised search. We use the PhysioNet Challenge 2021 dataset and represent the 12-lead ECGs as 12-channel inputs to match a vision transformer search space. We compare the performance of a NAS-derived architecture against a conventional, manually designed convolutional baseline model with and without an attention mechanism.

## Methods

### Dataset

We use the PhysioNet 2021 dataset for the NAS-driven arrhythmia classification. The PhysioNet Challenge 2021 ECG recordings originate from seven institutions across three continents (America, Asia, and Europe). The publicly available dataset includes 88,253 ECGs with 133 diagnoses or classes. Of these 133 classes, only 30 are used for evaluation. The choice of those 30 arrhythmias is justified by their clinical frequency and relevance, as well as their likelihood of being recognisable from ECG recordings [23]. The mean age of patients in the dataset is 59.7 ± 21.7 years. The sex distribution is 43.5% female and 56.5% male.

### Deep learning architecture

We base our deep learning architecture on Natarajan et al. [24]. The network architecture comprises a “clinical” and a “raw signal” feature transformer for multi-label ECG classification. The “clinical” features comprise the 20 most critical cardiac biomarkers (such as heart rate or RR interval) selected by an initial random forest model from more than 300 ECG features. The “raw signal” features are directly extracted from the ECG waveform. The “raw signal” learned features are passed to a transformer head. After the transformer head, we combine the “clinical” and “raw signal” features into the final fully connected layer.

The baseline models are based on the PhysioNet Challenge 21 winner [25]. The deep learning architecture is a residual neural network with a multi-head attention mechanism. The input is initially passed through a convolution layer with a kernel size of 15 and a stride of 2. The embedding dimension has an output of 256 and undergoes batch normalisation and a leaky Rectified Linear Unit activation. The subsequent five convolution layers have a kernel size of 9 and a stride of two. The convolution layer’s output follows a dropout layer (0.5) and is connected to the multi-head attention mechanism, with 8 heads on the 256-channel data. An attention visualisation highlights the ECG morphology that the deep learning architecture sees as critical for arrhythmia classification. An adaptive max pooling layer downsamples each of the 256 feature maps to a single maximum value. We use the same architecture but deactivate the attention mechanism for the baseline model without attention.

We chose the PhysioNet Challenge metric as the primary performance metric. The challenge metric converts the multi-label classification problem (i.e., multiple labels per ECG) into a multi-class evaluation system (i.e., one primary condition per ECG). The challenge metric gives full credit for correct classifications and partial credit for incorrect classifications, with closely related conditions receiving more credit than those that are entirely unrelated [23]. The area under the receiver operating characteristic curve (AUROC) is our secondary performance metric. Training was performed on a single NVIDIA L40S GPU. The model was trained for 50 epochs, and the best-performing epoch was selected for validation performance.

### Preprocessing

We filter the ECG signal with a bandpass Finite Impulse Response filter (bandwidth 3 Hz to 45 Hz, order 150) for noise reduction. Then, we apply min-max normalisation to constrain the lead recordings to the range −1–1. The min-max normalisation is distribution-agnostic and preserves the relative importance of values in the data. We resample all ECG recordings to 500 Hz and set the sequence length to 15s. Sequence lengths shorter than 15s were zero-padded, whilst a 15s window was taken out of longer signals.

We divide the ECG signal into uniform patches of 0.05s (25 points at 500 Hz) to ensure compatibility with the vision transformer architecture. We chose the patch size to be smaller than the QRS complex duration (≤ 0.10s in adults), the fastest-changing component of the cardiac waveform, to ensure we capture rapid ventricular transitions during time-series ECG-to-transformer-required patch-format conversion [26]. The preprocessing for the baseline models is based on Nejedly et al. [25].

We split the publicly available dataset into 80% for NAS training, 10% for validation, and 10% for testing and employ iterative stratification to ensure equal distribution of cardiac abnormalities across folds [27]. The 80% NAS training dataset is further split into a 50% NAS training set and a 50% NAS validation set following the DARTS search methodology proposed by Liu, Simonyan, and Yang [28].

### Search space

A NAS consists of a search space, a search strategy, and a performance estimation metric [29]. Four search spaces exist: Macro, cell, hierarchical, and chain-structured. Macro search spaces explore a large number of architectures [29]. Cell-based search spaces exhibit limited variance in predictive capability, regardless of their design [30]. Hierarchical search spaces pose significant technical implementation challenges [29]. A chain-structured search space provides a suitable compromise between computational feasibility and architectural variation.

We utilise the “AutoFormer” chain-structured search space from the Neural Network Intelligence (NNI). AutoFormer addresses self-attention blocks and convolutional layers and is therefore well-suited to our network’s architecture [18]. The AutoFormer search space can be subdivided into macro and microarchitecture search spaces, where parameters involving macro architecture design choices are more memory-intensive than those involving micro architecture design choices (see Fig 1).

**Fig 1:**
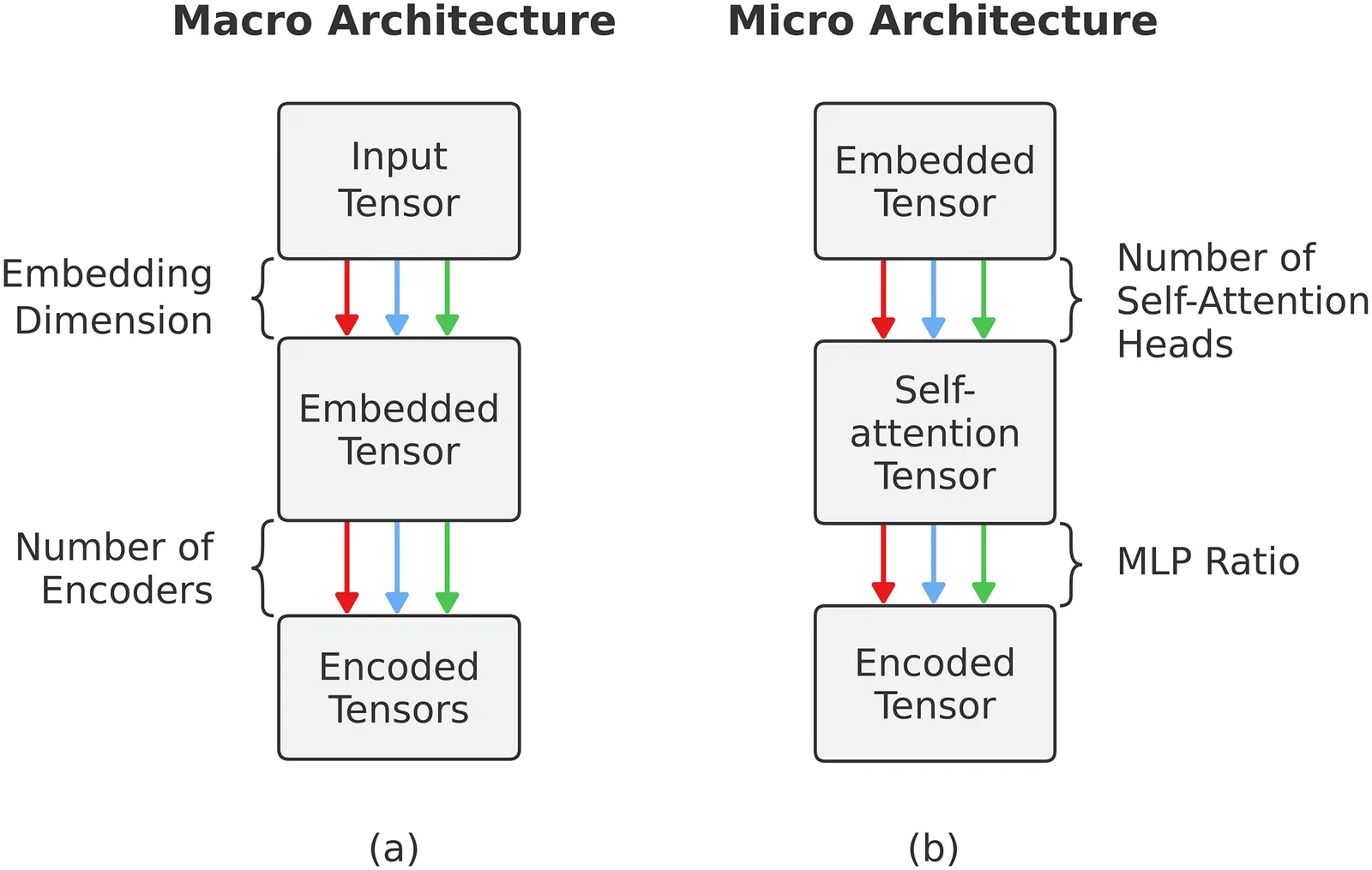
Relaxed directed acyclic graph of the (a) macro architecture search space and (b) micro search space. Grey cells indicate nodes, and the lines between cells represent edges. The different edge colours correspond to the available design options. Annotations exist between cells to indicate the overarching design choice encapsulated by the edge.

We construct a search space comprising 85.9 million parameters. For the Macro architecture parameters, we searched the embedding dimension (384, 768, 1,024), of the input vector and the network’s depth (4, 6, 8), i.e., the number of encoders. The number of self-attention heads (4, 6, 8)  in an encoder layer, the associated Query, Key, and Value (Q-K-V) dimensions (fixed), and the Multi-Layer Perceptron (MLP) ratio (1, 2, 4) within an encoder are the microarchitecture search parameters. Deeper vision transformers could have significantly increased the risk of overfitting, given that the PhysioNet Challenge 21 dataset is 1.3, 14, and 303 million times smaller than the standard training set sizes for vision-based transformers such as ImageNet-21k, ImageNet, and JFT, respectively [31,32].

The AutoFormer search space was developed for image classification and carries no inductive bias towards any specific dataset [18]. We therefore rearrange the one-dimensional ECG into a 12-lead image (i.e., a two-dimensional image). The vision transformer separates input images into patches and embeds them as vectors using either convolutional or linear operations. We add the positional and class encodings to the embedded vectors before passing them into a conventional transformer encoder block. The output from the encoder blocks is passed to a classification block [32].

### Search strategy

Black-box algorithms offer simpler implementations without compromising search robustness. At the same time, these algorithms are computationally demanding [29,33,34]. A one-shot strategy provides a suitable compromise between computational feasibility and search robustness.

We utilise the Differentiable ARchitecTure Search (DARTS) to explore an AutoFormer search space, as it was the most computationally feasible option available in the NNI package. DARTS transforms the discrete decision search space into a continuous, differentiable one through a process known as “relaxation” (see S1 Appendix for more information).

### Performance estimation

We use the standard binary cross-entropy (BCE) loss function as the primary performance metric for the NAS search, along with an additional elementary macro-accuracy measure.

### Hyperparameter optimisation

We perform hyperparameter optimisation on the NAS output using the PhysioNet 2020 dataset (a subset of the PhysioNet 2021 dataset). We remove any overlapping ECGs between the PhysioNet 2020 dataset and the validation or test folds of the 2021 dataset. We split the PhysioNet 2020 dataset for hyperparameter optimisation into 80% training, 10% validation, and 10% test folds using iterative stratification [27]. The hyperparameters we optimise are the input patch sizes (0.05, 0.1, and 0.2) and the loss function type (class weights, focal loss, and asymmetric loss (ASL); see the S1 Appendix for more information).

We also investigate the inclusion of three sets of clinical features as inputs to the deep model. The first set utilises the output of a random forest model that identified the 20 most important morphological, clinical, and statistical features present in a lead II ECG recording [24]. The second set contains demographic data regarding the patient’s age and gender. The third set is a combination of the first two sets.

## Results

Our deep learning architecture achieved an AUROC of 0.95 and a higher challenge metric of 0.66 during validation (see Table 1). During testing, the results were 0.97 for AUROC and 0.72 for the challenge metric. The winner of the PhysioNet Challenge 21 achieved a validation challenge score of 0.63 [25]*.* We ran the PhysioNet Challenge 21 winner and achieved an AUROC of 0.91 and a challenge metric of 0.69 during testing (the PhysioNet Challenge 21 winners also reported a challenge metric of 0.69 [25]). The version without the attention mechanism achieved an AUROC of 0.90 and the same challenge metric results on the same testing dataset.

**Table 1:** Overview of performance metrics of the different models. Abbreviations: Area under the receiver operating characteristic (AUROC); neural architecture search (NAS).

Performance metric	PhysioNet	PhysioNet without attention	Our NAS model	PhysioNet NAS
AUROC	0.91	0.90	0.97	0.91
Challenge metric	0.69	0.69	0.72	0.71

The best scores for individual arrhythmias were Normal Sinus Rhythm, Sinus Tachycardia, and Right Bundle Branch Block, with AUROCs of 0.99. Low-QRS voltage, T-wave abnormality, and T-wave inversion were the worst-performing arrhythmias, with AUROCs of 0.89, 0.90, and 0.92, respectively.

Fig 2 shows the NAS using the DARTS search strategy on the AutoFormer search space over 50 epochs. Each line represents an architecture evaluated during the search, with colour indicating validation accuracy (blue for higher accuracy, red for lower). The visualisation shows architectural parameters (embedding dimension, network depth, and block-specific choices for self-attention heads and MLP ratios) alongside validation loss and accuracy. We display only the first 4 encoder blocks since the search rarely selected deeper networks. We excluded parameter values that were never chosen during the search (e.g., E4 with 6 or 8 heads) from the visualisation. The optimal architecture uses an embedding dimension of 384, a depth of 4 encoder blocks, 4 self-attention heads, and an MLP ratio of 1.

**Fig 2:**
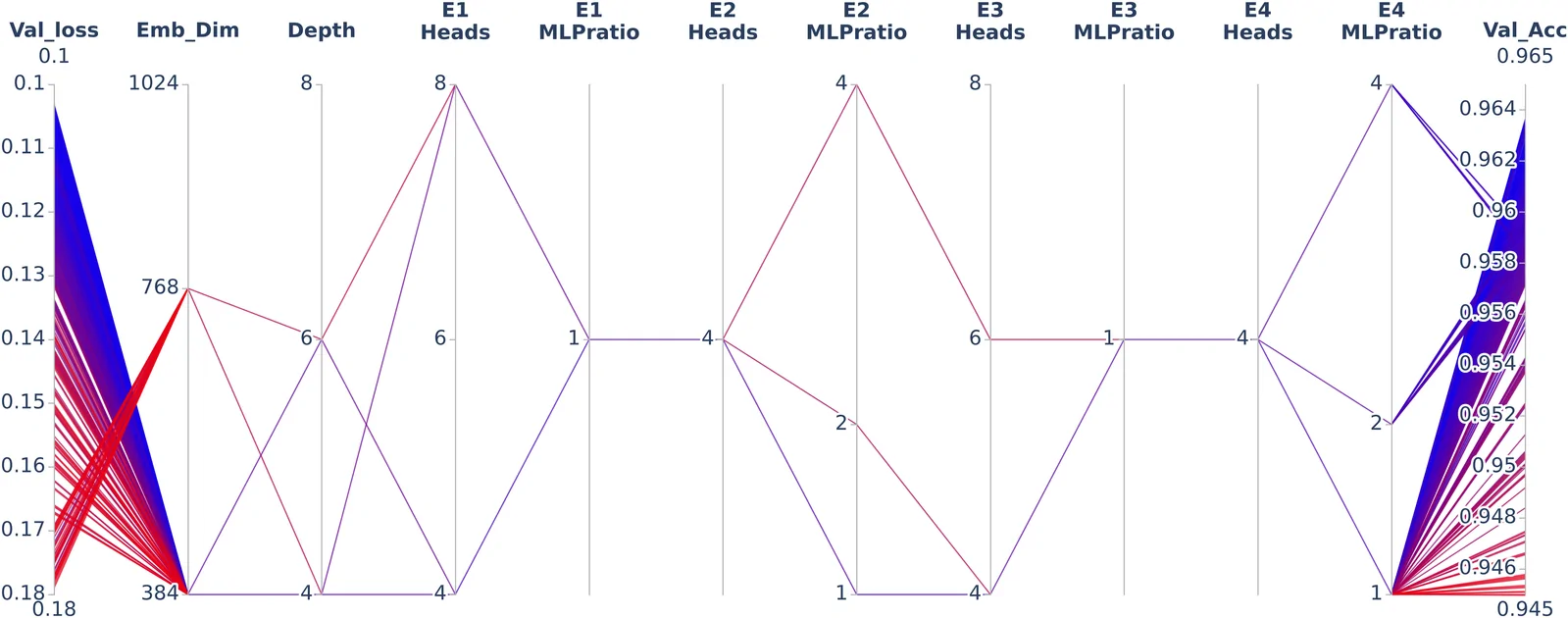
Parallel coordinates visualisation of NAS using DARTS on the AutoFormer search space over 50 epochs. Each line represents an architecture evaluated during the search, with colour indicating validation accuracy (blue for higher, red for lower). The visualisation shows architectural parameters (embedding dimension, network depth, and encoder block configurations including self-attention heads and MLP ratios) alongside validation loss and accuracy. Only the first four encoder blocks (E1-E4) are displayed, as deeper networks were rarely selected. Parameter values never chosen during the search were excluded from the visualisation.

We ran the PhysioNet Challenge 21 winner with only 4 self-attention heads, which was the optimal result from the NAS, and achieved an AUROC of 0.91 and a challenge metric of 0.71 (against 0.69 in the original PhysioNet Challenge architecture with 8 self-attention heads). Figs 3 and 4 show the attention mechanism visualisation comparison between the original (8 self-attention heads) and the NAS (4 self-attention heads) PhysioNet Challenge architecture for PVCs and LQT.

**Fig 3:**
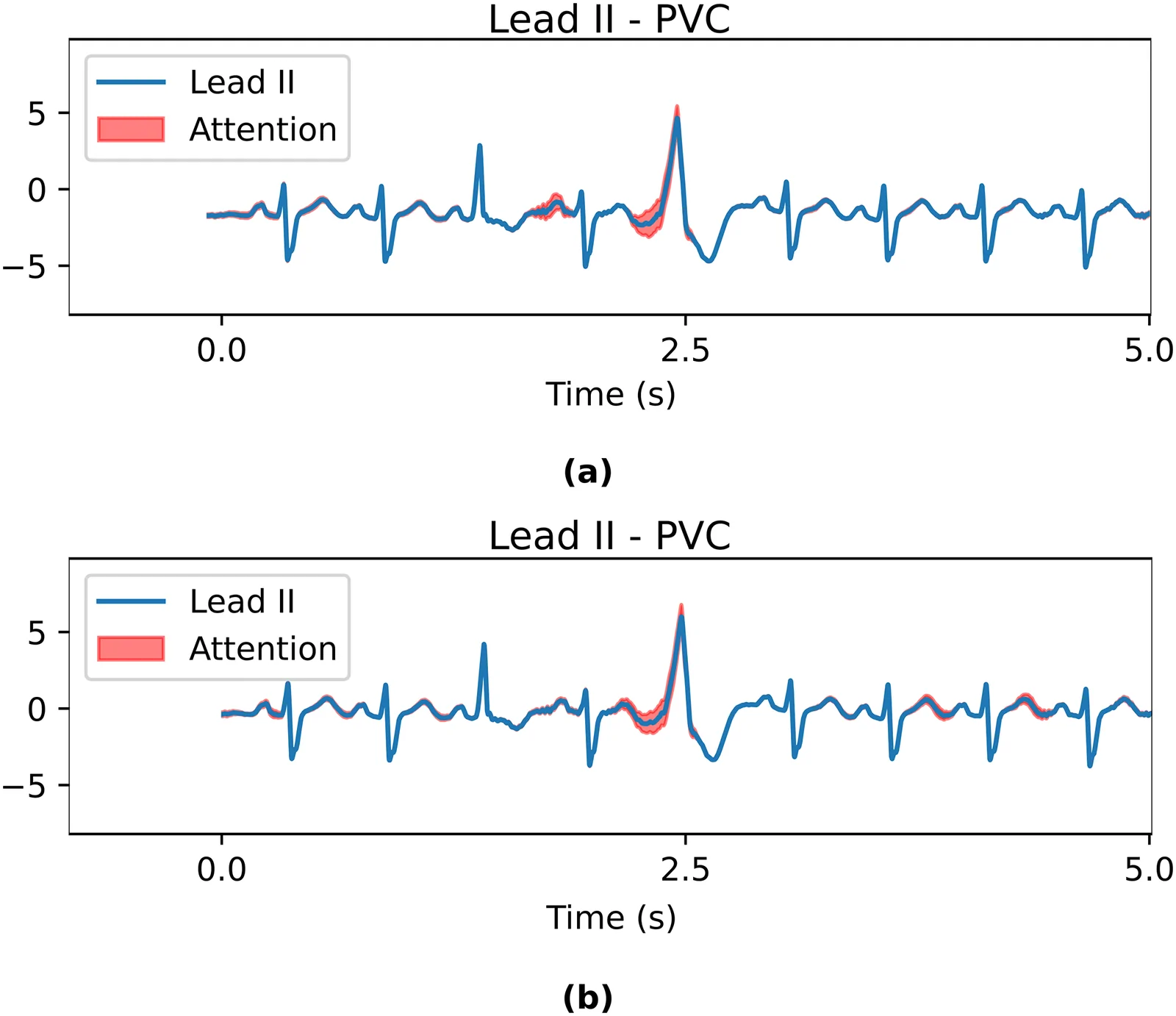
Plot of attention mechanism visualisations for PVCs. Comparison of attention mechanisms showing a PVC of lead II between (a) the original PhysioNet Challenge 21 architecture with 8 self-attention heads, and (b) the NAS PhysioNet Challenge architecture with 4 self-attention heads. Both architectures identify the PVC just before 2.5 seconds. The attention mechanism in (a) highlights the P-wave of the prior cycle, while (b) focuses additionally on two T-waves after the 4^th^ second. The y-axis is normalised and hence unitless. The patient (JS23661) is an 88-year-old male. Further diagnoses on the ECG are left anterior fascicular block, sinus tachycardia, poor R-wave progression, and fusion beats. Abbreviations: Premature ventricular contraction (PVC).

**Fig 4:**
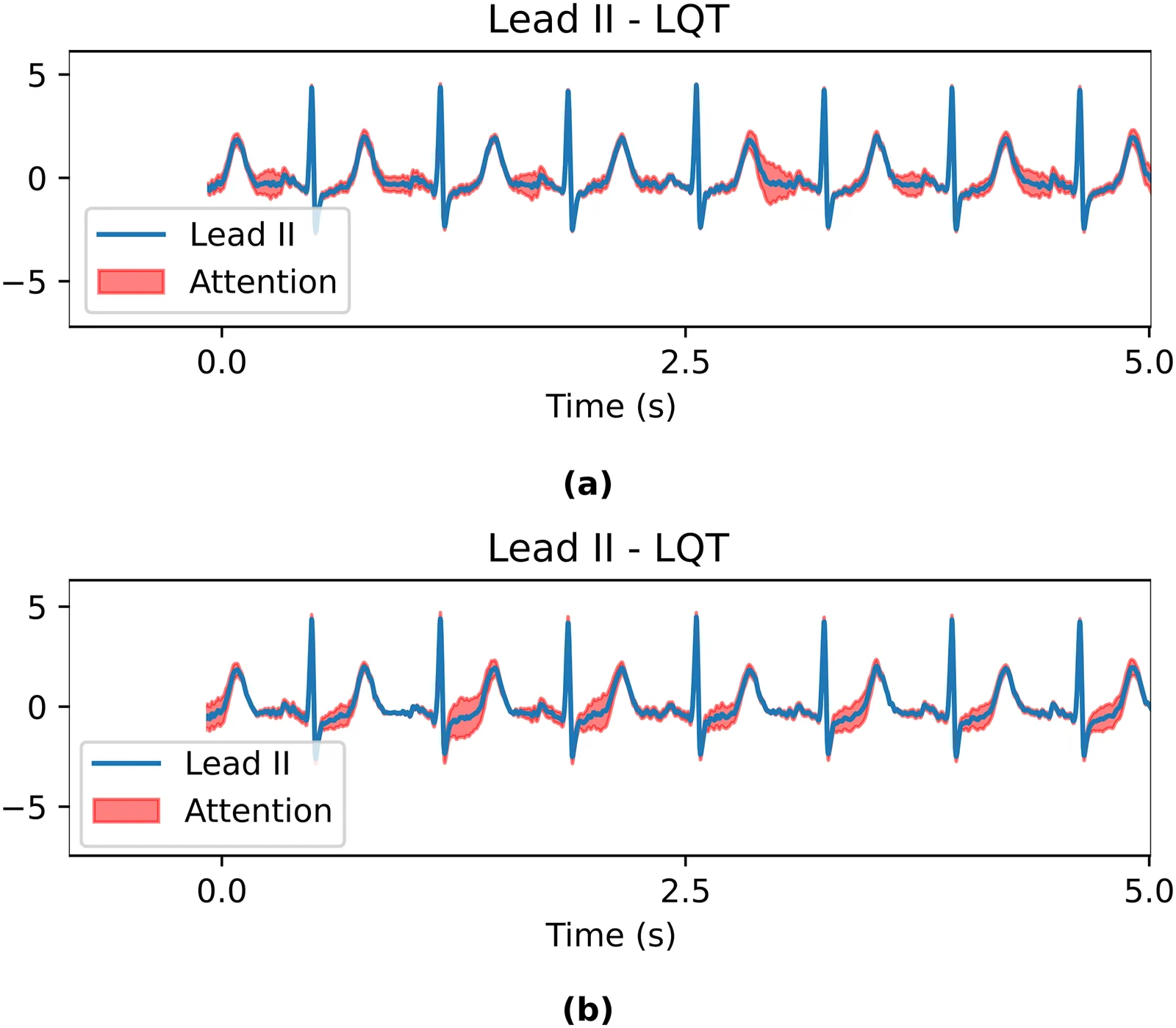
Plot of attention mechanism visualisations for LQT. Comparison of attention mechanisms showing an LQT of lead II between (a) the original PhysioNet Challenge 21 architecture with 8 self-attention heads, and (b) the NAS PhysioNet Challenge architecture with 4 self-attention heads. The attention mechanism in (a) mainly highlights the TP segment, while (b)’s main focus is on the ST segment. The y-axis is normalised and hence unitless. The patient (E02775) is a 24-year-old female. Further diagnoses on the ECG are abnormalities in the ST interval and T-wave. Abbreviations: Prolonged QT interval (LQT).

Fig 5 shows the hyperparameter optimisation results using parallel coordinates plots. Each line represents a hyperparameter configuration, with colour indicating the challenge metric score (blue for higher scores, red for lower). The plots display hyperparameter values (patch size, clinical features, loss function, weighted classes, and weight initialisation) alongside validation performance (challenge metric and AUROC). The optimal configuration used a patch size of 25, no clinical features, BCE loss without class weighting, and Xavier initialisation, achieving an AUROC of 0.91 and challenge metric of 0.5.

**Fig 5:**
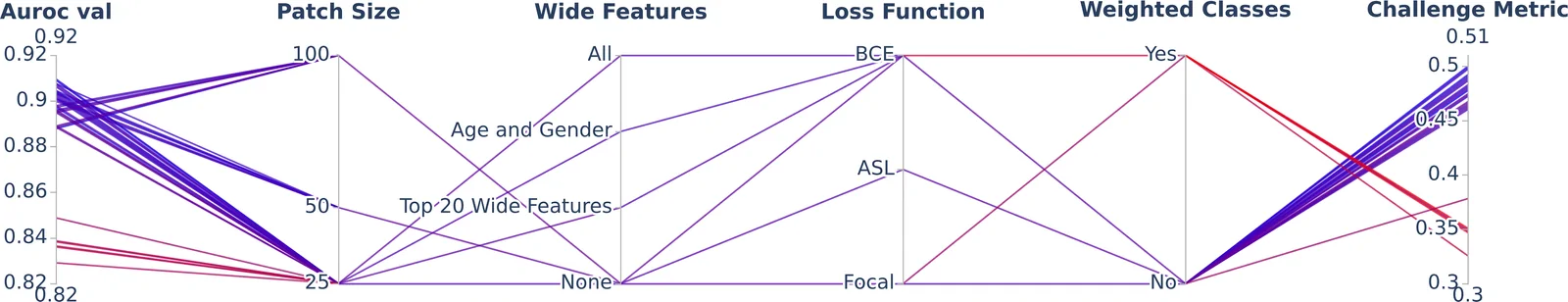
Parallel coordinates visualisation of hyperparameter optimisation results. Each line represents a hyperparameter configuration, with colour indicating challenge metric score (blue for higher, red for lower). The visualisation shows the hyperparameter values for patch size, clinical features (here called wide features as in [24]), loss function, and weighted classes alongside the validation performance metrics AUROC and the challenge metric.

## Discussion

### Neural architecture search

We apply NAS to design a deep learning architecture for arrhythmia classification. Early NAS iterations prefer larger architectures, as indicated by an embedding dimension of 768 and depth of 6. However, as the search progresses, both macro and micro architecture parameters converge towards minimal configurations: an embedding dimension of 384, a depth of 4 blocks, 4 self-attention heads, and an MLP ratio of 1. Since DARTS has no preference for minimising architectural parameters, the convergence towards minimal configurations indicates these perform better at arrhythmia classification with ECGs.

The convergence towards minimal architectural configurations suggests that effective AI-based ECG analysis does not require large computational resources, making deployment in resource-constrained clinical settings feasible. We hypothesise that downsampling and compact representations better capture the relevant features and potentially reveal redundancy in the ECG signal for transformer-based architectures. To test our hypothesis, we trained the PhysioNet Challenge 21 architecture without an attention head and another with only 4 self-attention heads, as the NAS proposed. The PhysioNet Challenge 21 architecture without an attention mechanism performed almost the same as the one with an attention mechanism (AUROC: 0.90vs 0.91; Challenge metric both 0.69). The PhysioNet Challenge 21 architecture with only 4 self-attention heads achieved the same AUROC but scored a higher challenge metric (0.71 vs 0.69) than the architecture with 8 self-attention heads, highlighting the advantage of minimal architectural configurations.

We compared the attention mechanism visualisations of the original PhysioNet Challenge 21 architecture (8 self-attention heads) and the NAS architecture (4 self-attention heads) for PVC and LQT. For PVC, both architectures detect the premature contraction. The attention mechanism with 8 self-attention heads additionally highlights the P-wave of the prior cycle, while the one with 4 self-attention heads focuses on two T-waves after the PVC. For LQT, the 8-headed attention mechanism primarily highlights the TP segment, whereas the 4-headed one primarily focuses on the clinically more relevant ST segment. In the literature, QT prolongation is frequently driven by a lengthened ST segment rather than the QRS complex itself [35].

### Hyperparameter optimisation

We perform hyperparameter optimisation (input patch size, weighted classes, and loss function) on the NAS output using the PhysioNet 2020 dataset. Variation in patch size (25–100 points, corresponding to 0.05 to 0.2 second intervals) has minimal impact on performance, as indicated by minor standard deviations across runs. Classification performance is similar whether model weights are included or excluded from the loss function. This similarity in performance suggests that the data are insufficient for the model to learn class-specific patterns independently.

Regarding loss functions, the BCE loss consistently outperforms the ASL loss. ASL uses a threshold parameter *m* to help models focus on complex cases by down-weighting evident ones. However, whilst *m* can improve focus on challenging examples, it may cause the model to overlook important borderline cases. Using the same threshold across all cardiac arrhythmias assumes equal diagnostic difficulty, which is clinically unrealistic. Without knowledge of the real-world prevalence distributions of arrhythmias, adequately tuning *m* is challenging. A fixed threshold choice likely hinders ASL performance in this context.

Hyperparameter optimisation indicates that class imbalance has the greatest impact on classification performance. None of the hyperparameter choices substantially improved classification performance for rare arrhythmias such as Low QRS Voltage (1.76% of the PhysioNet Challenge 21 dataset) and T-wave inversion (3.24%). These results suggest that class imbalance fundamentally limits classification performance. Data imbalance is particularly problematic for transformer architectures, which lack inductive biases and require extensive training datasets. Convolutional neural networks, for example, allow clinicians and researchers to encode domain knowledge directly into the architecture, such as kernel sizes that reflect the temporal scale of clinically relevant ECG features. Transformer architectures offer no equivalent constraint, learning what to attend to entirely from the data. On an imbalanced dataset, this means the model may preferentially learn features of common classes at the expense of rare ones.

Although the lack of inductive bias makes transformers sensitive to imbalance, the transformer’s self-attention mechanism can learn abstract clinical representations autonomously. This ability is evident since clinical features do not improve classification performance. We hypothesise that the self-attention mechanism may already have learned abstract representations of these features (e.g., heart rate variability from temporal patterns), thereby simplifying clinical deployment by eliminating the need for an explicit feature-extraction pipeline.

## Limitations

The primary limitation of our study is the dataset’s class imbalance. Despite extensive hyperparameter optimisation, we observe no significant improvement in classification performance, with specific rare cardiac abnormalities, such as Low QRS Voltage (1.76%) and T-wave inversion (3.24%), proving particularly difficult to classify.

Although selected for their computational feasibility, popularity, and compatibility with our transformer-based architecture, both AutoFormer and DARTS were originally developed for image classification tasks. As a result, representing the one-dimensional ECG as a two-dimensional image to conform to the AutoFormer search space may not optimally preserve the signal’s temporal characteristics. This includes applying the NAS-configured self-attention heads to the PhysioNet Challenge 21 architecture. Nonetheless, reducing the number of self-attention heads increased the challenge metric, showing that the NAS results themselves translate, in this case, from an image classification task to a 1D time-signal task.

We want to note that this study is a proof of concept. We chose to run the NAS on an existing 2D vision search space (AutoFormer) because no comparable and dedicated search space exists for native 1D convolutional neural network architectures. We aimed first to establish whether the NAS approach identifies meaningful simplifications at all before investing in building a dedicated 1D search space.

## Conclusion

Our NAS revealed that minimal architectural configurations outperform larger alternatives for ECG-based arrhythmia classification. Both macro and micro-level parameters converged toward the smallest values in the search space. The convergence towards minimal architectural configurations suggests that effective AI-based ECG analysis does not require substantial computational resources, making deployment in resource-constrained clinical settings feasible. Given that SCD is sudden and unexpected, prevention strategies in free-living environments require lightweight computational resources suitable for wearable devices. The key insight is that architectural simplicity, rather than complexity, drives classification performance for cardiac abnormalities from ECGs, as adding complexity risks overfitting without improving generalisation. To confirm that architectural simplicity improves performance in ECGs rather than reflecting a search space that does not translate well to the final architecture, we also reduced the number of attention heads in the original PhysioNet Challenge 8-head architecture to 4, as suggested by the NAS. This reduced-head architecture achieved a higher challenge metric while also correctly highlighting both PVC and LQT in the attention visualisations.

Hyperparameter optimisation indicates that class imbalance fundamentally limits classification performance, as none of the hyperparameter choices substantially improved classification performance for rare arrhythmias such as Low QRS Voltage and T-wave inversion. Transformer architectures lack inductive biases, making them particularly sensitive to class imbalance. However, the self-attention mechanism can autonomously abstract clinical representations, simplifying clinical deployment by eliminating the need for an explicit feature-extraction pipeline.

Future work could explore custom search spaces tailored to clinical ECG applications, such as incorporating domain-specific signal-processing operations or multi-scale temporal features and investigate how different NAS-derived architectures affect model explainability.

## Supporting information

S1 Appendix Supporting methodological details on Neural Architecture Search (DARTS) and loss function formulation. This file contains mathematical equations used for the differentiable architecture search and loss function.
(DOCX)
