# Supplementary material for: Deep learning optimisation for cardiology: Neural Architecture Search-driven arrhythmia classification with electrocardiograms
Source: PLoS One. 2026 Nov 6;21(11):e0362129. doi: 10.1371/journal.pone.0362129 (PMC13742905; doi:10.1371/journal.pone.0362129)
Supplement: Supplementary file 1 [file pone.0362129.s001.docx]

S1 Appendix

Deep learning optimisation for cardiology: Neural Architecture Search-driven arrhythmia classification with electrocardiograms

**Erik Vanegas Müller^ab^*, Arese Joe-Oshodi^b^, Abhirup Banerjee^bc^ and Mauricio Villarroel^ab^***

^a^The Podium Institute for Sports Medicine and Technology, Department of Engineering Science, University of Oxford, Oxford, United Kingdom
^b^Institute of Biomedical Engineering, Department of Engineering Science, University of Oxford, Oxford, United Kingdom
^c^Division of Cardiovascular Medicine, Radcliffe Department of Medicine, University of Oxford, Oxford, United Kingdom

Correspondence email: erik.vanegasmuller@eng.ox.ac.uk; mauricio.villarroel@eng.ox.ac.uk

DARTS

The search space is first converted into a directed acyclic graph with corresponding nodes and edges (1). In the relaxed AutoFormer search space, nodes include the embedding vector, the multi-head self-attention, and the input and output tensors. Edges refer to the operations applied between the tensors, such as the embedding tensor's dimension, the number of encoders to stack in the transformer, the MLP ratio, and the number of self-attention heads within the encoder block. DARTS uses a user-defined loss function (binary cross-entropy in our case) for evaluation on both the training and validation sets.

To create the super network that encodes all possible architecture variations, all potential edges $\sigma_{m}^{(i,j)}$ that can be applied between nodes $X^{(i)}$ are multiplied by associated architecture parameters $\alpha_{m}^{(i,j)}$. The sum of these weighted edge operations creates a mixed operation variable between the two defined nodes $X^{(i)}$ and $X^{(j)}$ [14].

$$\sigma_{m}^{(i,j)}=\sum_{m=1}^{k} \frac{e^{\alpha_{m}^{(i,j)}}}{\sum_{n=1}^{l} e^{\alpha_{n}^{(i,j)}}}\sigma_{m}^{(i,j)}$$

(1)

The supernetwork is then defined as the combination of the nodes and mixed operations between them:

$$Supernetwork=\left\{ \sigma^{(i,j)},X^{(i)} \right\}$$

(2)

The architectural parameters are continuous and encode all possible discrete network design choices. Therefore, by optimising the weights with respect to a chosen evaluation metric, we effectively optimise the network architecture. Operations that perform better on the selected evaluation metric will have higher weightings in the mixed-value variable. At the end of the search, an argmax is performed over the mixed operation variable, using the updated architectural parameters to select the final discrete edge operation (2).

$$\sigma_{m}^{(i,j)}=\arg\max_{\alpha^{(i,j)}} \sigma^{(i,j)}$$

(3)

Traditional network weights ω must be trained simultaneously with the architectural weights. Therefore, the search is a bi-level optimisation problem of minimising the validation loss L_val_ over the architectural weights. The optimisation is subject to finding optimal network weights ω∗ that minimise the training loss L_train_ given the architectural parameters (2).

|  | $\min_{\alpha} L_{\mathrm{val}}(\omega^{*}(\alpha),\alpha)$ | (4) |
| --- | --- | --- |

so that,

$$\omega^{*}\left( \alpha\right)=\arg\min_{\omega} L_{\mathrm{train}}(\omega,\alpha)$$

(5)

Due to the nested formulation of the architectural loss function, which includes optimising network weights, obtaining an analytical gradient solution is both technically and computationally demanding. Therefore, an approximation of the optimal weights for the current architectural parameters is used in place of *ω*^∗^, and the weights are jointly optimised with the architectural parameters during gradient descent.

$$\omega^{*}\left( \alpha\right)=\omega-\epsilon\nabla_{\omega}L_{\mathrm{train}}(\omega,\alpha)$$

(6)

Conducting the bi-level optimisation approximation follows traditional gradient descent algorithms highlighted in Liu, Simonyan, and Yang (2). DARTS randomly initialises the supernetwork with associated mixed operations and nodes that have not converged.
1. Architectural gradient descent to update $\alpha$ parameters.

$$\alpha=\alpha-\eta\nabla_{\alpha}L_{\mathrm{val}}(\omega-\epsilon\nabla_{\omega}L_{\mathrm{train}}\left( \omega,\alpha\right),\alpha)$$

(7)

2. Network gradient descent to update $\omega^{*}$ weights.

$$\omega^{*}\left( \alpha\right)=\omega-\epsilon\nabla_{\omega}L_{\mathrm{train}}(\omega,\alpha)$$

(8)

DARTS applies argmax over the mixed operations to select the final discrete edges and associated architecture. Then DARTS retrains the final architecture on the entire dataset to obtain optimised architecture-specific network weights.

Loss function details

A general definition of standard loss functions used in multi-label classification is given in eq. (9) where the total loss $L_{\mathrm{total}}$ is calculated over the sum of *k* samples. The individual sample losses are calculated based on contributions from optimistic predictions ($L_{+}$) and negative predictions ($L_{-}$), given label *y*.

Focal loss utilises a focusing parameter *γ* to mitigate the impact of easily classified negative or positive samples on the loss function. Known as a soft threshold, this parameter allows the model to focus on difficult-to-classify classes highlighted in eq. (12) and eq. (13). Building upon this, asymmetric loss introduces separate focusing parameters for positive *γ*_+_ and negative samples *γ*_−_ with an additional shifted probability parameter that is set to 0 if it is smaller than a pre-defined threshold parameter *t*, given in eq. (11). A combination of these two factors allows ASL to retain a soft threshold whilst introducing a hard threshold, setting the impact of very easily classified negative samples to 0.

$$L_{\mathrm{total}}=\sum_{i=1}^{k} L_{\mathrm{sample}}$$

(9)

with

$$L_{\mathrm{sample}}=-yL_{+}-(1-y)L_{-}$$

(10)

$$p_{t}=\max(p-t,0)$$

(11)

$$Focal \left\{ \begin{aligned} L_{+}={(1-p)}^{\gamma}\log p \\ L_{-}=p_{t}^{\gamma}\log(1-p^{t}) \end{aligned} \right.$$

(12)

$$ASL \left\{ \begin{aligned} L_{+}={(1-p)}^{\gamma+}\log p \\ L_{-}=p_{t}^{\gamma-}\log(1-p^{t}) \end{aligned} \right.$$

(13)

We prioritised parameter variation in asymmetric loss (ASL) over focal loss because it has been reported to achieve better classification performance on benchmark imbalanced datasets. Optimising the hyperparameters of the ASL function was challenging in itself, as it depends on three separate parameters. The loss function was only a part of our wider optimisation experiments; therefore, for the sake of time and project completion, we constrained the parameter space of the ASL function to include only the hard threshold parameter *m*. This constraint had the most significant impact on the loss function; therefore, the γ+ and γ− parameters were chosen in line with best-practice heuristics for multi-class datasets.

References

1.	Yao X, Qiu Y, Li X, editors. A Survey on One-shot Neural Architecture Search. IOP Conference Series Materials Science and Engineering; 2020: IOP Publishing.

2.	Liu H, Simonyan K, Yang Y. DARTS: Differentiable Architecture Search. ArXiv. 2018;abs/1806.09055.
