# Supplementary material for: Deep learning optimisation for cardiology: Neural Architecture Search-driven arrhythmia classification with electrocardiograms
Source: PLoS One. 2026 Nov 6;21(11):e0362129. doi: 10.1371/journal.pone.0362129 (PMC13742905; doi:10.1371/journal.pone.0362129)
Supplement: Supplementary file 2 [file pone.0362129.s003.docx]

Deep learning optimisation for cardiology: Neural Architecture Search-driven arrhythmia classification with electrocardiograms

Response to reviewers

# Comments to the Author

1. Is the manuscript technically sound, and do the data support the conclusions?

The manuscript must describe a technically sound piece of scientific research with data that supports the conclusions. Experiments must have been conducted rigorously, with appropriate controls, replication, and sample sizes. The conclusions must be drawn appropriately based on the data presented.

Reviewer #1: Partly

Reviewer #2: Partly

Answer:

We appreciate the reviewers' feedback. Although the reviewers' comments were substantial, we believe they helped make this manuscript more technically sound. Reviewer 2 asked us to compare the results of the NAS-configured neural network with those of a simple neural network without an attention mechanism. We performed this test, the NAS-configured model still performed better in terms of AUROC (0.97 vs 0.91) and the challenge metric (0.72 vs 0.69).

To demonstrate that simplicity drives performance, we conducted a second experiment. The PhysioNet Challenge 21 Winner, the baseline performance model we compared our NAS model against, is an 8-head convolutional neural network. The NAS-configured model suggested 4 heads as optimal. Therefore, we trained the PhysioNet Challenge 21 Winner with 4 heads, resulting in the same AUROC (0.69 vs 0.69) but an improved challenge metric (0.71 vs 0.69) compared to the original PhysioNet Challenge architecture.

Reviewer 1 asked us to include attention visualisation. We included attention visualisation for two arrhythmias that can lead to sudden cardiac death, namely premature ventricular contractions and prolonged QT intervals. We compared the attention visualisations of the arrhythmias produced by the original 8-headed and our NAS-improved 4-headed PhysioNet Challenge 21 architecture. Both architectures identified the premature ventricular contraction. For the prolonged QT interval, the 4-headed architecture focuses on the clinically relevant segment, whereas the 8-headed architecture does not.

2. Has the statistical analysis been performed appropriately and rigorously?

Reviewer #1: No

Reviewer #2: No

Answer:

We thank the reviewers for this assessment. We note that our evaluation follows the standard methodology of the PhysioNet/CinC Challenge, using the official challenge metric and AUROC as defined by the challenge organisers. We followed the same protocol used to score the original 2021 challenge submission. We did not perform statistical significance testing since standard tests assume independent observation. The performance scores obtained through cross-validation are not independent due to overlapping training sets.

3. Have the authors made all data underlying the findings in their manuscript fully available?

The [PLOS Data policy](http://track.editorialmanager.com/CL0/http:%2F%2Fwww.plosone.org%2Fstatic%2Fpolicies.action%23sharing/1/010f019f5eb1c267-42067276-a2e0-48a7-8751-cef352122764-000000/RhMqIzMArjGDPdPzxKvB38wHT3HNMPI_iRL9dbSz3kI=258) requires authors to make all data underlying the findings described in their manuscript fully available without restriction, with rare exception (please refer to the Data Availability Statement in the manuscript PDF file). The data should be provided as part of the manuscript or its supporting information, or deposited to a public repository. For example, in addition to summary statistics, the data points behind means, medians and variance measures should be available. If there are restrictions on publicly sharing data—e.g. participant privacy or use of data from a third party—those must be specified.

Reviewer #1: Yes

Reviewer #2: No

Answer:

We thank the reviewers for their comments. All the datasets we used (PhysioNet2020 and PhysioNet2021) are open-source and available at [https://moody-challenge.physionet.org/2020/](https://moody-challenge.physionet.org/2021/) and <https://moody-challenge.physionet.org/2021/>, respectively. We completed the data availability statement, including the PhysioNet 2020 challenge (see marked-up manuscript pg. 23 lines 483–485).

4. Is the manuscript presented in an intelligible fashion and written in standard English?

PLOS ONE does not copyedit accepted manuscripts, so the language in submitted articles must be clear, correct, and unambiguous. Any typographical or grammatical errors should be corrected at revision, so please note any specific errors here.

Reviewer #1: Yes

Reviewer #2: No

Answer:

We appreciate that the reviewers took the time to evaluate the English language in the script. We went through every sentence and corrected grammar and spelling mistakes. Where necessary, we rewrote sentences for greater intelligibility.

# Review Comments to the Author

Please use the space provided to explain your answers to the questions above. You may also include additional comments for the author, including concerns about dual publication, research ethics, or publication ethics. (Please upload your review as an attachment if it exceeds 20,000 characters)

# Reviewer 1

1. Provide a dedicated comparison with recent NAS and Vision Transformer approaches.

Answer:

We appreciate the comment. We included recent NAS and Vision Transformer approaches in the Introduction (see pg. 7. lines 141–148 and 156–165 on the marked-up manuscript). The introduction now reads:

Transformers are a popular choice of deep learning models which introduced the “self-attention” concept (14). Originally proposed for a translation task, self-attention assigns each element of a sequence a query, key, and value. Multi-head self-attention mechanisms therefore allow for simultaneous focus on different relationships within a sequence. The number of attention heads is a central architectural parameter for transformer performance (15). The architecture's design is problem-dependent; it depends on the researchers' prior knowledge and experience (16,17). Other important transformer parameters include the embedding dimension, the number of encoders, and the multi-layer perceptron (MLP) ratio (18).

The design choices for deep learning architectures are not necessarily interchangeable across different applications. The field of neural architecture search (NAS) aims to automate the construction of deep learning architectures, thereby reducing potential biases introduced by traditional manual architecture design and hyperparameter fine-tuning (17,19). So et al. (20) used an evolutionary NAS to discover a Transformer variant that outperformed the original model on a machine translation task. Chen et al. (21) applied a one-shot NAS to Vision Transformers, finding architectures that outperformed original model designs while using fewer parameters. Wu et al. (22) applied a differentiable NAS to Vision Transformers, thereby reducing parameter count while outperforming the original deep learning model. Azarmehr et al. (23) designed a NAS to classify anatomical echocardiographic views, reducing trainable parameters by up to 99.9% while maintaining comparable classification performance.

2. Include detailed information regarding search space design, computational cost, search duration, hardware resources, and convergence criteria.

Answer:

We thank the reviewer for the comment. The detailed description of the search space design is contained in the Methodology section (pp. 12–13, lines 258–287 on the marked-up manuscript). We also included a detailed description of the search strategy “DARTS” in the Appendix. We added the hardware resources (pg. 11, lines 237–238 on the marked-up script) and convergence criteria (pg. 11, lines 238–239 on the marked-up script).

Unfortunately, we cannot report the computational cost or search duration because we run computations on a shared university cluster, where computational resources are allocated based on the cluster’s demand.

We added the following information on pg. 11, lines 237–239, on the marked-up copy of the manuscript:

Training was performed on a single NVIDIA L40S GPU. The model was trained for 50 epochs, and the best-performing epoch was selected as validation performance.

3. Include attention visualization

Answer:

Thank you for this suggestion. We added an attention visualisation for two arrhythmias which can lead to sudden cardiac death, namely premature ventricular beats and prolonged QT intervals. Please see figures 3 and 4 on pages 17 and 18 of the marked-up script, respectively.

4. Consider external validation on independent ECG datasets to demonstrate robustness and generalizability.

Answer:

We thank the reviewer for the suggestion. The PhysioNet Challenge 21 dataset is already a combination of several different independent datasets. These datasets stem from 7 different institutions across 3 continents. The entire dataset contains over 100 different arrhythmias, of which 26 are evaluated. Testing our AI model on a different dataset would require delving deeper into the arrhythmias of the external validation dataset to explain why the performance metrics differ. We see the value in NAS in optimising the neural network towards a given dataset. We aim to underscore the importance of dataset-specific optimisation, which is not routinely practised.

5. Report model size, parameter count, inference latency, and energy consumption metrics.

Answer:

Thank you for your comment. We reported the model size and parameter. Please refer to pg. 13, lines 274–281, in the marked-up manuscript. We revised the methods section to describe the methodology that we used to construct a search space comprising 85.9 million parameters. For the Macro architecture parameters, we searched the embedding dimension (384, 768, 1,024) of the input vector and the network’s depth (4, 6, 8), i.e., the number of encoders. The number of self-attention heads (4, 6, 8) in an encoder layer, the associated Query, Key, and Value (Q-K-V) dimensions (fixed), and the Multi-Layer Perception (MLP) ratio (1, 2, 4) within an encoder are the microarchitecture search parameters.

We cannot capture energy consumption or inference latency metrics because, as explained in reply to comment 2, we compute on a shared cluster, where computational resources are allocated according to user demand.

6. Minor grammatical revisions and sentence restructuring would improve readability.

Answer:

Thank you for pointing this out. We have now revised the manuscript thoroughly, improving grammar and readability.

# Reviewer 2

1. The biggest issue I have is with the core methodological setup: forcing a 1D, 12-lead ECG signal into a pseudo-2D "image" so it fits an off-the-shelf vision search space (AutoFormer) and search strategy (DARTS) never built for this kind of data. The authors acknowledge this in their limitations, but acknowledging a problem isn't the same as addressing it.

I'd want to see this NAS-derived model benchmarked against at least a simple native 1D-CNN or 1D-transformer baseline, not just the fixed 2020 challenge winner, before I believe "architectural simplicity drives performance" as opposed to "this particular search space doesn't transfer well to ECGs regardless of size."

Several conclusions, particularly that architectural simplicity drives performance and that self-attention autonomously learns clinical representations, are stated too strongly and are not fully supported by the experimental evidence.

Answer:

We thank the reviewer for this feedback. We agree with his comments. We made substantial changes to our manuscript. Please refer to pg. 20 lines 394–408, pg. 22 lines 449–456, and pg. 23 lines 466–470 on the marked-up manuscript to review these major changes.

We compared our NAS-derived model against the same PhysioNet Challenge 21 winner architecture without an attention mechanism. The NAS-configured model still performed better in both AUROC (0.97 vs 0.90) and the challenge metric (0.72 vs 0.69).

We then went a step further. The NAS search identified 4 self-attention heads as optimal. Our goal was to see whether the results of an image classification task translate to a 1D-CNN. We applied this result to the PhysioNet Challenge 21 winner architecture (originally built with 8 heads) and found that the challenge metric improved (0.71 vs 0.69) at the same AUROC (0.91). These results support our conclusion that architectural simplicity drives performance.

To support the claim that self-attention autonomously learns clinical representations, we plotted a self-attention visualisation of premature ventricular contractions (PVCs) and prolonged QT interval (LQT). Both the 8-head and 4-head architectures correctly localised the PVC. For LQT, the 4-head architecture focused on a more clinically relevant morphology (ST-segment) compared to the 8-head architecture (TP-segment).

We want to note that this study is a proof of concept. We chose to run the NAS on an existing 2D vision search space (AutoFormer) because no comparable and dedicated search space exists for native 1D CNN architectures. We aimed first to establish whether the NAS approach identifies meaningful simplifications at all before investing in building a dedicated 1D search space.
