# Supplementary material for: Deep learning optimisation for cardiology: Neural Architecture Search-driven arrhythmia classification with electrocardiograms
Source: PLoS One. 2026 Nov 6;21(11):e0362129. doi: 10.1371/journal.pone.0362129 (PMC13742905; doi:10.1371/journal.pone.0362129)
Supplement: Peer review history

# Author comment: Author response to Decision Letter 0

**DOI:** 10.1371/journal.pone.0362129.r001

**Transfer Alert**

This paper was transferred from another journal. As a result, its full editorial history (including decision letters, peer reviews and author responses) may not be present.

23 Mar 2026

# Peer review: Decision Letter 0

**DOI:** 10.1371/journal.pone.0362129.r002

**Authors:** Ibrahim Marai

13 Jul 2026

Dear Dr. Vanegas Müller,

Please submit your revised manuscript by Aug 27 2026 11:59PM. If you will need more time than this to complete your revisions, please reply to this message or contact the journal office at plosone@plos.org. When you're ready to submit your revision, log on to https://www.editorialmanager.com/pone/ and select the 'Submissions Needing Revision' folder to locate your manuscript file.

- A letter that responds to each point raised by the academic editor and reviewer(s). You should upload this letter as a separate file labeled 'Response to Reviewers'.
- A marked-up copy of your manuscript that highlights changes made to the original version. You should upload this as a separate file labeled 'Revised Manuscript with Track Changes'.
- An unmarked version of your revised paper without tracked changes. You should upload this as a separate file labeled 'Manuscript'.

If you would like to make changes to your financial disclosure, please include your updated statement in your cover letter. Guidelines for resubmitting your figure files are available below the reviewer comments at the end of this letter.

If applicable, we recommend that you deposit your laboratory protocols in protocols.io to enhance the reproducibility of your results. Protocols.io assigns your protocol its own identifier (DOI) so that it can be cited independently in the future. For instructions see: https://journals.plos.org/plosone/s/submission-guidelines#loc-laboratory-protocols. Additionally, PLOS ONE offers an option for publishing peer-reviewed Lab Protocol articles, which describe protocols hosted on protocols.io. Read more information on sharing protocols at https://plos.org/protocols?utm_medium=editorial-email&utm_source=authorletters&utm_campaign=protocols.

As the corresponding author, your ORCID iD is verified in the submission system and will appear in the published article. PLOS supports the use of ORCID, and we encourage all coauthors to register for an ORCID iD and use it as well. Please encourage your coauthors to verify their ORCID iD within the submission system before final acceptance, as unverified ORCID iDs will not appear in the published article. *Only*  the individual author can complete the verification step; PLOS staff *cannot*  verify ORCID iDs on behalf of authors.

We look forward to receiving your revised manuscript.

Kind regards,

Ibrahim Marai, MD

Academic Editor

PLOS One

Journal Requirements:

When submitting your revision, we need you to address these additional requirements.

1. Please ensure that your manuscript meets PLOS ONE's style requirements, including those for file naming. The PLOS ONE style templates can be found at

https://journals.plos.org/plosone/s/file?id=wjVg/PLOSOne_formatting_sample_main_body.pdf and

https://journals.plos.org/plosone/s/file?id=ba62/PLOSOne_formatting_sample_title_authors_affiliations.pdf

2.  Please note that PLOS One has specific guidelines on code sharing for submissions in which author-generated code underpins the findings in the manuscript. In these cases, we expect all author-generated code to be made available without restrictions upon publication of the work. Please review our guidelines at https://journals.plos.org/plosone/s/materials-and-software-sharing#loc-sharing-code and ensure that your code is shared in a way that follows best practice and facilitates reproducibility and reuse.

3. Thank you for stating the following financial disclosure:

“EV and MV are funded by The Podium Institute of Sports Medicine and Technology. AB is supported by the Royal Society University Research Fellowship (Grant No. URFR1’314).  “

Please state what role the funders took in the study. If the funders had no role, please state: "The funders had no role in study design, data collection and analysis, decision to publish, or preparation of the manuscript."

If this statement is not correct you must amend it as needed.

Please include this amended Role of Funder statement in your cover letter; we will change the online submission form on your behalf.

4. Please include captions for your Supporting Information files at the end of your manuscript, and update any in-text citations to match accordingly. Please see our Supporting Information guidelines for more information: http://journals.plos.org/plosone/s/supporting-information.

5. If the reviewer comments include a recommendation to cite specific previously published works, please review and evaluate these publications to determine whether they are relevant and should be cited. There is no requirement to cite these works unless the editor has indicated otherwise.

Reviewers' comments:

Reviewer's Responses to Questions

**Comments to the Author**

1. Is the manuscript technically sound, and do the data support the conclusions?

Reviewer #1: Partly

Reviewer #2: Partly

**********

2. Has the statistical analysis been performed appropriately and rigorously?

Reviewer #1: No

Reviewer #2: No

**********

3. Have the authors made all data underlying the findings in their manuscript fully available?

The PLOS Data policy

Reviewer #1: Yes

Reviewer #2: No

**********

4. Is the manuscript presented in an intelligible fashion and written in standard English?

Reviewer #1: Yes

Reviewer #2: No

**********

Reviewer #1: 1. Provide a dedicated comparison with recent NAS and Vision Transformer approaches.

2. Include detailed information regarding search space design, computational cost, search duration, hardware resources, and convergence criteria.

3. Include attention visualization

4. Consider external validation on independent ECG datasets to demonstrate robustness and generalizability.

5. Report model size, parameter count, inference latency, and energy consumption metrics.

6. Minor grammatical revisions and sentence restructuring would improve readability.

Reviewer #2: The biggest issue I have is with the core methodological setup: forcing a 1D, 12-lead ECG signal into a pseudo-2D "image" so it fits an off-the-shelf vision search space (AutoFormer) and search strategy (DARTS) never built for this kind of data. The authors acknowledge this in their limitations, but acknowledging a problem isn't the same as addressing it.

I'd want to see this NAS-derived model benchmarked against at least a simple native 1D-CNN or 1D-transformer baseline, not just the fixed 2020 challenge winner, before I believe "architectural simplicity drives performance" as opposed to "this particular search space doesn't transfer well to ECGs regardless of size."

Several conclusions, particularly that architectural simplicity drives performance and that self-attention autonomously learns clinical representations, are stated too strongly and are not fully supported by the experimental evidence.

**********

what does this mean?). If published, this will include your full peer review and any attached files.

If you choose “no”, your identity will remain anonymous but your review may still be made public.

**Do you want your identity to be public for this peer review?**  For information about this choice, including consent withdrawal, please see our Privacy Policy

Reviewer #1: No

Reviewer #2: No

**********

[NOTE: If reviewer comments were submitted as an attachment file, they will be attached to this email and accessible via the submission site. Please log into your account, locate the manuscript record, and check for the action link "View Attachments". If this link does not appear, there are no attachment files.]

To ensure your figures meet our technical requirements, please review our figure guidelines: https://journals.plos.org/plosone/s/figures

You may also use PLOS’s free figure tool, NAAS, to help you prepare publication quality figures: https://journals.plos.org/plosone/s/figures#loc-tools-for-figure-preparation.

NAAS will assess whether your figures meet our technical requirements by comparing each figure against our figure specifications.

# Author comment: Author response to Decision Letter 1

**DOI:** 10.1371/journal.pone.0362129.r003

7 Oct 2026

Deep learning optimisation for cardiology: Neural Architecture Search-driven arrhythmia classification with electrocardiograms

Response to reviewers

Comments to the Author

1. Is the manuscript technically sound, and do the data support the conclusions?

The manuscript must describe a technically sound piece of scientific research with data that supports the conclusions. Experiments must have been conducted rigorously, with appropriate controls, replication, and sample sizes. The conclusions must be drawn appropriately based on the data presented.

Reviewer #1: Partly

Reviewer #2: Partly

Answer:

We appreciate the reviewers' feedback. Although the reviewers' comments were substantial, we believe they helped make this manuscript more technically sound. Reviewer 2 asked us to compare the results of the NAS-configured neural network with those of a simple neural network without an attention mechanism. We performed this test, the NAS-configured model still performed better in terms of AUROC (0.97 vs 0.91) and the challenge metric (0.72 vs 0.69).

To demonstrate that simplicity drives performance, we conducted a second experiment. The PhysioNet Challenge 21 Winner, the baseline performance model we compared our NAS model against, is an 8-head convolutional neural network. The NAS-configured model suggested 4 heads as optimal. Therefore, we trained the PhysioNet Challenge 21 Winner with 4 heads, resulting in the same AUROC (0.69 vs 0.69) but an improved challenge metric (0.71 vs 0.69) compared to the original PhysioNet Challenge architecture.

Reviewer 1 asked us to include attention visualisation. We included attention visualisation for two arrhythmias that can lead to sudden cardiac death, namely premature ventricular contractions and prolonged QT intervals. We compared the attention visualisations of the arrhythmias produced by the original 8-headed and our NAS-improved 4-headed PhysioNet Challenge 21 architecture. Both architectures identified the premature ventricular contraction. For the prolonged QT interval, the 4-headed architecture focuses on the clinically relevant segment, whereas the 8-headed architecture does not.

2. Has the statistical analysis been performed appropriately and rigorously?

Reviewer #1: No

Reviewer #2: No

Answer:

We thank the reviewers for this assessment. We note that our evaluation follows the standard methodology of the PhysioNet/CinC Challenge, using the official challenge metric and AUROC as defined by the challenge organisers. We followed the same protocol used to score the original 2021 challenge submission. We did not perform statistical significance testing since standard tests assume independent observation. The performance scores obtained through cross-validation are not independent due to overlapping training sets.

3. Have the authors made all data underlying the findings in their manuscript fully available?

The PLOS Data policy requires authors to make all data underlying the findings described in their manuscript fully available without restriction, with rare exception (please refer to the Data Availability Statement in the manuscript PDF file). The data should be provided as part of the manuscript or its supporting information, or deposited to a public repository. For example, in addition to summary statistics, the data points behind means, medians and variance measures should be available. If there are restrictions on publicly sharing data—e.g. participant privacy or use of data from a third party—those must be specified.

Reviewer #1: Yes

Reviewer #2: No

Answer:

We thank the reviewers for their comments. All the datasets we used (PhysioNet2020 and PhysioNet2021) are open-source and available at https://moody-challenge.physionet.org/2020/ and https://moody-challenge.physionet.org/2021/, respectively. We completed the data availability statement, including the PhysioNet 2020 challenge (see marked-up manuscript pg. 23 lines 482–484).

4. Is the manuscript presented in an intelligible fashion and written in standard English?

PLOS ONE does not copyedit accepted manuscripts, so the language in submitted articles must be clear, correct, and unambiguous. Any typographical or grammatical errors should be corrected at revision, so please note any specific errors here.

Reviewer #1: Yes

Reviewer #2: No

Answer:

We appreciate that the reviewers took the time to evaluate the English language in the script. We went through every sentence and corrected grammar and spelling mistakes. Where necessary, we rewrote sentences for greater intelligibility.

Review Comments to the Author

Please use the space provided to explain your answers to the questions above. You may also include additional comments for the author, including concerns about dual publication, research ethics, or publication ethics. (Please upload your review as an attachment if it exceeds 20,000 characters)

Reviewer 1

1. Provide a dedicated comparison with recent NAS and Vision Transformer approaches.

Answer:

We appreciate the comment. We included recent NAS and Vision Transformer approaches in the Introduction (see pg. 7. lines 140–147 and 155–164 on the marked-up manuscript). The introduction now reads:

Transformers are a popular choice of deep learning models which introduced the “self-attention” concept (14). Originally proposed for a translation task, self-attention assigns each element of a sequence a query, key, and value. Multi-head self-attention mechanisms therefore allow for simultaneous focus on different relationships within a sequence. The number of attention heads is a central architectural parameter for transformer performance (15). The architecture's design is problem-dependent; it depends on the researchers' prior knowledge and experience (16,17). Other important transformer parameters include the embedding dimension, the number of encoders, and the multi-layer perceptron (MLP) ratio (18).

The design choices for deep learning architectures are not necessarily interchangeable across different applications. The field of neural architecture search (NAS) aims to automate the construction of deep learning architectures, thereby reducing potential biases introduced by traditional manual architecture design and hyperparameter fine-tuning (17,19). So et al. (20) used an evolutionary NAS to discover a Transformer variant that outperformed the original model on a machine translation task. Chen et al. (21) applied a one-shot NAS to Vision Transformers, finding architectures that outperformed original model designs while using fewer parameters. Wu et al. (22) applied a differentiable NAS to Vision Transformers, thereby reducing parameter count while outperforming the original deep learning model. Azarmehr et al. (23) designed a NAS to classify anatomical echocardiographic views, reducing trainable parameters by up to 99.9% while maintaining comparable classification performance.

2. Include detailed information regarding search space design, computational cost, search duration, hardware resources, and convergence criteria.

Answer:

We thank the reviewer for the comment. The detailed description of the search space design is contained in the Methodology section (pp. 12–13, lines 257–286 on the marked-up manuscript). We also included a detailed description of the search strategy “DARTS” in the Appendix. We added the hardware resources (pg. 11, lines 236–237 on the marked-up script) and convergence criteria (pg. 11, lines 237–238 on the marked-up script).

Unfortunately, we cannot report the computational cost or search duration because we run computations on a shared university cluster, where computational resources are allocated based on the cluster’s demand.

We added the following information on pg. 11 lines 236–238 on the marked-up copy of the manuscript:

Training was performed on a single NVIDIA L40S GPU. The model was trained for 50 epochs, and the best-performing epoch was selected as validation performance.

3. Include attention visualization

Answer:

Thank you for this suggestion. We added an attention visualisation for two arrhythmias which can lead to sudden cardiac death, namely premature ventricular beats and prolonged QT intervals. Please see figures 3 and 4 on pages 17 and 18 of the marked-up script, respectively.

4. Consider external validation on independent ECG datasets to demonstrate robustness and generalizability.

Answer:

We thank the reviewer for the suggestion. The PhysioNet Challenge 21 dataset is already a combination of several different independent datasets. These datasets stem from 7 different institutions across 3 continents. The entire dataset contains over 100 different arrhythmias, of which 26 are evaluated. Testing our AI model on a different dataset would require delving deeper into the arrhythmias of the external validation dataset to explain why the performance metrics differ. We see the value in NAS in optimising the neural network towards a given dataset. We aim to underscore the importance of dataset-specific optimisation, which is not routinely practised.

5. Report model size, parameter count, inference latency, and energy consumption metrics.

Answer:

Thank you for your comment. We reported the model size and parameter. Please refer to pg. 13 lines 273–280on the marked-up manuscript. We revised the methods section to describe the methodology that we used to construct a search space comprising 85.9 million parameters. For the Macro architecture parameters, we searched the embedding dimension (384, 768, 1,024) of the input vector and the network’s depth (4, 6, 8), i.e., the number of encoders. The number of self-attention heads (4, 6, 8) in an encoder layer, the associated Query, Key, and Value (Q-K-V) dimensions (fixed), and the Multi-Layer Perception (MLP) ratio (1, 2, 4) within an encoder are the microarchitecture search parameters.

We cannot capture energy consumption or inference latency metrics because, as explained in reply to comment 2, we compute on a shared cluster, where computational resources are allocated according to user demand.

6. Minor grammatical revisions and sentence restructuring would improve readability.

Answer:

Thank you for pointing this out. We have now revised the manuscript thoroughly, improving grammar and readability.

Reviewer 2

1. The biggest issue I have is with the core methodological setup: forcing a 1D, 12-lead ECG signal into a pseudo-2D "image" so it fits an off-the-shelf vision search space (AutoFormer) and search strategy (DARTS) never built for this kind of data. The authors acknowledge this in their limitations, but acknowledging a problem isn't the same as addressing it.

I'd want to see this NAS-derived model benchmarked against at least a simple native 1D-CNN or 1D-transformer baseline, not just the fixed 2020 challenge winner, before I believe "architectural simplicity drives performance" as opposed to "this particular search space doesn't transfer well to ECGs regardless of size."

Several conclusions, particularly that architectural simplicity drives performance and that self-attention autonomously learns clinical representations, are stated too strongly and are not fully supported by the experimental evidence.

Answer:

We thank the reviewer for this feedback. We agree with his comments. We made substantial changes to our manuscript. Please refer to pg. 20 lines 393–407, pg. 22 lines 448–455, and pg. 23 lines 465–469 on the marked-up manuscript to review these major changes.

We compared our NAS-derived model against the same PhysioNet Challenge 21 winner architecture without an attention mechanism. The NAS-configured model still performed better in both AUROC (0.97 vs 0.90) and the challenge metric (0.72 vs 0.69).

We then went a step further. The NAS search identified 4 self-attention heads as optimal. Our goal was to see if the result for an image classification task translates to a 1D-CNN. We applied this result to the PhysioNet Challenge 21 winner architecture (originally built with 8 heads) and found that the challenge metric improved (0.71 vs 0.69) at the same AUROC (0.91). These results support our conclusion that architectural simplicity drives performance.

To support the claim that self-attention autonomously learns clinical representations, we plotted a self-attention visualisation of premature ventricular contractions (PVCs) and prolonged QT interval (LQT). Both the 8-head and 4-head architectures correctly localised the PVC. For LQT, the 4-head architecture focused on a more clinically relevant morphology (ST-segment) compared to the 8-head architecture (TP-segment).

We want to note that this study is a proof of concept. We chose to run the NAS on an existing 2D vision search space (AutoFormer) because no comparable and dedicated search space exists for native 1D CNN architectures. We aimed first to establish whether the NAS approach identifies meaningful simplifications at all before investing in building a dedicated 1D search space.

Attachment Submitted filename: Vanegas_Response_to_Reviewers_Plos_One_v4_2026_10_05.docx

# Editor report: Decision Letter 1

**DOI:** 10.1371/journal.pone.0362129.r004

**Authors:** Ibrahim Marai

20 Oct 2026

Deep learning optimisation for cardiology: Neural Architecture Search-driven arrhythmia classification with electrocardiograms

PONE-D-26-14263R1

Dear Dr. Erik Vanegas Müller

We’re pleased to inform you that your manuscript has been judged scientifically suitable for publication and will be formally accepted for publication once it meets all outstanding technical requirements.

Within one week, you’ll receive an e-mail detailing the required amendments. When these have been addressed, you’ll receive a formal acceptance letter and your manuscript will be scheduled for publication.

An invoice will be generated when your article is formally accepted. Please note, if your institution has a publishing partnership with PLOS and your article meets the relevant criteria, all or part of your publication costs will be covered. Please make sure your user information is up-to-date by logging into Editorial Manager at Editorial Manager® and clicking the ‘Update My Information' link at the top of the page. For questions related to billing, please contact billing support.

If your institution or institutions have a press office, please notify them about your upcoming paper to help maximize its impact. If they’ll be preparing press materials, please inform our press team as soon as possible -- no later than 48 hours after receiving the formal acceptance. Your manuscript will remain under strict press embargo until 2 pm Eastern Time on the date of publication. For more information, please contact onepress@plos.org.

Kind regards,

Ibrahim Marai, MD

Academic Editor

PLOS One

# Editor report: Acceptance letter

**DOI:** 10.1371/journal.pone.0362129.r005

**Authors:** Ibrahim Marai

PONE-D-26-14263R1

PLOS One

Dear Dr. Vanegas Müller,

I'm pleased to inform you that your manuscript has been deemed suitable for publication in PLOS One. Congratulations! Your manuscript is now being handed over to our production team.

At this stage, our production department will prepare your paper for publication. This includes ensuring the following:

* All references, tables, and figures are properly cited

* All relevant supporting information is included in the manuscript submission,

* There are no issues that prevent the paper from being properly typeset

You will receive further instructions from the production team, including instructions on how to review your proof when it is ready. Please keep in mind that we are working through a large volume of accepted articles, so please give us a few days to review your paper and let you know the next and final steps.

Lastly, if your institution or institutions have a press office, please let them know about your upcoming paper now to help maximize its impact. If they'll be preparing press materials, please inform our press team within the next 48 hours. Your manuscript will remain under strict press embargo until 2 pm Eastern Time on the date of publication. For more information, please contact onepress@plos.org.

You will receive an invoice from PLOS for your publication fee after your manuscript has reached the completed accept phase. If you receive an email requesting payment before acceptance or for any other service, this may be a phishing scheme. Learn how to identify phishing emails and protect your accounts at https://explore.plos.org/phishing.

If we can help with anything else, please email us at customercare@plos.org.

Thank you for submitting your work to PLOS One and supporting open access.

Kind regards,

PLOS One Editorial Office Staff

on behalf of

Dr. Ibrahim Marai

Academic Editor

PLOS One
